# KRAS^G12C^ inhibition using MRTX1257: a novel radio-sensitizing partner

**DOI:** 10.1186/s12967-023-04619-0

**Published:** 2023-10-31

**Authors:** Pierre-Antoine Laurent, Marina Milic, Clément Quevrin, Lydia Meziani, Winchygn Liu, Daphné Morel, Nicolas Signolle, Céline Clémenson, Antonin Levy, Michele Mondini, Eric Deutsch

**Affiliations:** 1 Department of Radiation Oncology, Gustave Roussy Cancer Campus, 114 rue Edouard Vaillant, 94805 Villejuif, France; 2 INSERM U1030, Molecular Radiation Therapy and Therapeutic Innovation, Gustave Roussy Cancer Campus, University of Paris-Saclay, 114 rue Edouard Vaillant, 94805 Villejuif, France; 3 SIRIC SOCRATE, Gustave Roussy, Villejuif, France; 4 Drug Development Department (DITEP), Gustave Roussy Cancer Campus, Villejuif, France; 5 Experimental and Translational Pathology Platform (PETRA), AMMICa, INSERM US23/UAR3655, Gustave Roussy Cancer Campus, Villejuif, France

**Keywords:** MRTX1257, Radiotherapy, KRAS inhibitors, Tumor, Radio-sensitizing, KRAS G12C, Immune microenvironment

## Abstract

**Background:**

*KRAS* activating mutations are considered the most frequent oncogenic drivers and are correlated with radio-resistance in multiple cancers including non-small cell lung cancer (NSCLC) and colorectal cancer. Although KRAS was considered undruggable until recently, several KRAS inhibitors have recently reached clinical development. Among them, MRTX849 (Mirati Therapeutics) showed encouraging clinical outcomes for the treatment of selected patients with *KRAS*^*G12C*^ mutated NSCLC and colorectal cancers. In this work, we explore the ability of MRTX1257, a KRAS^G12C^ inhibitor analogous to MRTX849, to radio-sensitize *KRAS*^*G12C*+*/*+^ mutated cell lines and tumors.

**Methods:**

Both in vitro and in vivo models of radiotherapy (RT) in association with MRTX1257 were used, with different *RAS* mutational profiles. We assessed in vitro the radio-sensitizing effect of MRTX1257 in CT26 KRAS^G12C+/+^, CT26 WT, LL2 WT and LL2 *NRAS* KO (LL2 NRAS^−/−^) cell lines. In vivo, we used syngeneic models of subcutaneous CT26 KRAS^G12C+/+^ tumors in BALB/c mice and T cell deficient athymic *nu/nu* mice to assess both the radio-sensitizing effect of MRTX1257 and its immunological features.

**Results:**

MRTX1257 was able to radio-sensitize CT26 KRAS^G12C+/+^ cells in vitro in a time and dose dependent manner. Moreover, RT in association with MRTX1257 in BALB/c mice bearing CT26 KRAS^G12C+/+^ subcutaneous tumors resulted in an observable cure rate of 20%. However, no durable response was observed with similar treatment in athymic nude mice. The analysis of the immune microenvironment of CT26 KRAS^G12C+/+^ tumors following RT and MRTX1257 showed an increase in the proportion of various cell subtypes including conventional CD4 + T cells, dendritic cells type 2 (cDC2) and inflammatory monocytes. Furthermore, the expression of PD-L1 was dramatically down-regulated within both tumor and myeloid cells, thus illustrating the polarization of the tumor microenvironment towards a pro-inflammatory and anti-tumor phenotype following the combined treatment.

**Conclusion:**

This work is the first to demonstrate in vitro as in vivo the radio-sensitizing effect of MRTX1257, a potent KRAS^G12C^ inhibitor compatible with oral administration, in CT26 KRAS^G12C^ mutated cell lines and tumors. This is a first step towards the use of new combinatorial strategies using KRAS inhibitors and RT in KRAS^G12C^ mutated tumors, which are the most represented in NSCLC with 14% of patients harboring this mutational profile.

**Supplementary Information:**

The online version contains supplementary material available at 10.1186/s12967-023-04619-0.

## Background

Radiotherapy (RT) plays a central role in cancer treatment. During the course of their treatment, it is estimated that nearly 50% of all cancer patients have indications for radiotherapy either with curative or palliative intent [1, 2].

The presence and type of activated oncogenes within tumor cells influence prognosis and therapeutic response, including RT. *RAS* (Rat sarcoma viral oncogene homolog) is considered the most frequently mutated oncogene with nearly 19% of cancer patients harboring a *RAS* mutation [3]. The *KRAS* (Kirsten rat sarcoma viral oncogene) isoform represents 75% of RAS mutant cancers and is therefore considered a crucial oncogene.

*KRAS* mutations occur in approximately 20–25% of non-small-cell lung cancers (NSCLC) [4], more than 80% of pancreatic cancers and 30% of colorectal and cholangial cancers [5]. When it comes to *KRAS*^*G12C*^ isoform mutations, they are estimated to occur in 14% of NSCLC adenocarcinomas [6] and 5% of colorectal cancers [7].

The ability of the *RAS* oncogene to drive radio-resistance has been extensively studied since the 1990’s. *RAS* mutations are long known to provide radiation resistance to tumor cells, as demonstrated first in mouse cell lines transformed with *HRAS*, *NRAS* or *KRAS* mutations [8, 9]. Upstream and downstream pathways from RAS, including EGFR expression, AKT phosphorylation, PI3K and MAPK signaling are also associated with the response to radiation [10, 11].

These results are in line with clinical observations in patients with *RAS* mutated cancers, often showing overall resistance to therapies and thus associated with poor outcomes [12–15]. Therefore, *RAS* mutations appear to be a target of interest, and different strategies of RAS inhibition using transfected rodent cells or human derived cell lines showed promising radio-sensitizing outcomes. These strategies involve farnesyltransferase inhibitors [16], lovastatin [17], prenyltransferase inhibitors [18], the transfection of anti-RAS single chain antibody fragment [19], or the use of an antisense vector [20].

Until recently, *KRAS* mutations were considered undruggable. Efforts to target KRAS directly have faced major difficulties owing to its pico-molar affinity for GTP/GDP and the absence of allosteric regulatory sites. *KRAS* oncogenic mutations result in functional activation of RAS family proteins by impairing GTP hydrolysis [21]. KRAS mutant proteins have a reduced capability to hydrolyze GTP or to interact with the GTPase-activating proteins, maintaining the oncogene and the downstream pathways constitutively activated. The lack of specific inhibitors targeting the KRAS hydrophobic pocket as well as the complexity of downstream pathways have contributed to the challenge of developing effective therapeutic agents.

Two specific KRAS^G12C^ inhibitors, sotorasib (AMG510, Amgen) and adagrasib (MRTX849, Mirati Therapeutics) recently earned the breakthrough designation by the US Food and Drug administration to treat metastatic lung cancer patients harboring *KRAS*^*G12C*^ mutations who have progressed on at least one systemic therapy. These small molecules are able to irreversibly inhibit KRAS^G12C^ through a unique interaction with the P2 pocket trapping KRAS^G12C^ in the inactive GDP-bound state similar to other allele-specific inhibitors [22].

Data from the registrational phase 1/2 KRYSTAL-1 trial (NCT03785249) reported results of a phase 2 cohort including 116 patients with metastatic *KRAS*^*G12C*^ mutated NSCLC treated with at least one prior systemic therapy before receiving adagrasib [6]. The objective response rate (ORR), progression-free survival (PFS) and median overall survival (OS) were respectively 42.9%, 6.5 months and 12.6 months. Despite these promising results, resistance often occurs in patients, paving the way for further strategies able to improve the long-term efficacy of KRAS^G12C^ inhibitors. Among these strategies, the association of KRAS^G12C^ inhibitors with focal RT is of interest.

RT is standard of care at all non-advanced inoperable NSCLC stages, including *KRAS*^*G12C*^ mutant tumors [23]. Therefore, demonstrating the benefit of an association between KRAS^G12C^ inhibitors and RT has the potential to make patients with *KRAS*^*G12C*^ mutations benefit from this new therapeutic strategy. Furthermore, *KRAS* is responsible of an immunosuppressive tumor microenvironment within mutated tumors in a PD-L1 dependent manner [24, 25], and the effect of MRTX849 was diminished when CT26 KRAS^G12C+/+^ were grown in T cell deficient nude mice [26]. This illustrates the necessity to consider the infiltrating immune cells in the studies assessing the therapeutic associations using KRAS inhibitors.

In this work, we explore the efficacy of the association between RT and MRTX1257, a selective and covalent KRAS^G12C^ inhibitor analogous to MRTX849, in animal models of cancer. Moreover, we show the different effects of the association of RT and MRTX1257 in reshaping the tumor immune microenvironment, i.e. the important down-regulation of PD-L1 in tumor and myeloid cells, as well as the increase of the infiltration of conventional CD4^+^ T cells, inflammatory monocytes and dendritic cells type 2 (cDC2).

## Material and methods

### Cell culture, chemicals and antibodies

Cell lines CT26 WT and LL2 WT were obtained from ATCC. CT26 harboring homozygous *KRAS*^*G12C*^ mutation (CT26 KRAS^G12C+/+^) and LL2 KO for *NRAS* (LL2 NRAS^−/−^) were provided by Mirati Therapeutics (USA).

CT26 KRAS^G12C+/+^ and CT26 WT cell lines were maintained in RPMI-1640 (Gibco) supplemented with 10% fetal calf serum and 1% penicillin–streptomycin (Gibco) and cultured at 37 °C with 5% CO2. LL2 WT and LL2 NRAS^−/−^ were maintained in DMEM + GlutaMAX + Pyruvate (Gibco) supplemented with 10% fetal calf serum and 1% penicillin–streptomycin (Gibco) and cultured at 37 °C with 5% CO2.

MRTX1257 was provided by Mirati Therapeutics and reconstituted using cyclodextrin Captisol (Ligand, San Diego, USA) in case of oral administration in mice or dimethylsulfoxide (DMSO) for in vitro experiments.

### Clonogenic survival assay

CT26 WT, CT26 KRAS^G12C+/+^, LL2 WT and LL2 NRAS^−/−^ were collected using trypsin–EDTA 0.05% (Gibco) and counted with Cellometer K2 Fluorescent cell counter (NEXCELOM, Massachussetts, USA) by trypan blue viability at least equal to 90%.

For CT26 WT and CT26 KRAS^G12C+/+^ cell lines, cells were seeded in triplicates at 50 to 400 cells/well (CT26 WT) or 100 to 1200 cells/well (CT26 KRAS^G12C+/+^) in 6-well plates during 12 h in 2 mL of culture medium. Three hours before irradiation, the culture medium was changed for the medium containing MRTX1257 at the indicated concentration. Twenty-four or 48 h after irradiation, the medium containing MRTX1257 was changed for drug-free medium.

For LL2 WT and LL2 NRAS^−/−^, 250 to 500 cells were seeded per well in 6-well plates during 3 h in 1 mL of Methocult M3134 (StemCell Technologies) supplemented in culture medium and containing MRTX1257 at the indicated dose. Twenty-four or 48 h after irradiation, a dilution of MRTX1257 by a factor 10 was done by adding 9 mL of drug-free culture medium to the 1 mL of Methocult M3134 in each well.

Plates were irradiated at the indicated dose using an X-RAD 320 irradiator (Precision X-Ray, USA). Seven days after irradiation, colonies equal or higher to 50 cells were counted. The number of colonies counted was normalized by the number of cells per well seeded at the start of the experiment in order to define the survival fraction. The different survival fractions were finally normalized by the survival fraction in the control non-irradiated condition for each concentration of MRTX1257 tested and then plotted in survival curves.

### Mouse models

Animal studies were conducted in compliance with all applicable regulations and guidelines of the Ethical approval Committee CCEA 26 at Gustave Roussy.

Seven-week-old BALB/c WT and athymic nude female mice were obtained from Janvier Labs (Le Genest-Saint-Isle, France) and housed in a pathogen-free facility. Mice were subcutaneously inoculated in their right flank with 10^6^ CT26 KRAS^G12C+/+^ cells or 5.10^5^ CT26 WT cells in 50µL of pH 7.2 phosphate-buffer saline (PBS) solution (Gibco).

Once the tumors reached an average volume of 90–100 mm^3^, estimated using the following formula Tumor volume = (length x width x width)/2*,* with length and width of tumors measured using a digital caliper, mice were randomized into the different treatment groups.

On the day of randomization (= D-1 before RT), as well as on D1 and D3 after RT, mice received an oral administration of MRTX1257 at the dose of 50 mg/kg reconstituted in 5 ml/kg of Captisol vehicle or the same volume of Captisol vehicle alone, according to the treatment group. At D0, mice randomized into RT only and combination groups received a single fraction of 6 Gy to the tumor volume using a Varian Tube NDI 226 (X-ray machine; 200 kV; tube current 15 mA; beam filter: 0.2 mmCu, dose-rate 1.15 Gy/minute). Radiation was only delivered to the tumor mass by using a custom shielding and an appropriate device to immobilize the mouse.

Tumor volumes were measured twice a week using a digital caliper and the previous formula. When tumor volumes reached 1200 mm^3^ or when any of critical points including weight loss > 20%, tumor necrosis or suffering appeared, mice were sacrificed.

BALB/c mice showing complete response of CT26 KRAS^G12C+/+^ tumors following irradiation combined with MRTX1257 were rechallenged with s.c. injection of either 10^6^ CT26 KRAS^G12C+/+^ cells or 5.10^5^ CT26 WT cells in contralateral flank. Tumor growth and survival were evaluated using the same methods than described above, and these data were compared with those of naive BALB/c mice receiving similar s.c. injections.

### Immunohistochemistry

The day after the last administration of MRTX1257 (= D4 after RT), animals were sacrificed by cervical dislocation and subcutaneous tumors were harvested and then fixated using paraformaldehyde (PFA) 4%.

For detection of cleaved CD8, 4 µm sections were processed for heat-induced antigen retrieval (ER2 corresponding EDTA buffer pH9) for 20 min at 100 °C. Slides were incubated with the antibody (clone D4W2Z #98,941, 1: 400, Cell Signaling) for 1 h at room temperature. Then, the slides were incubated with the Rabbit HRP PowerVision Kit (Leica Biosystems, #PV6119). The signal was revealed with diaminobenzidine (DAB).

For Ki67, slides were incubated with a monoclonal rabbit anti-Ki67 antibody (Cell Signaling, 1: 500). The signal was revealed with the Rabbit PowerVision Kit (UltraVision Technologies).

All slides were scanned at 20X using VS120 scanner (Olympus, Japan). Images were processed and staining quantified using QuPath v0.4.0 [27]. Regions of interest were manually delineated. Inside these regions, tissue was automatically detected using a trained classifier. Color deconvolution (Hematoxylin, DAB and residual) was applied to the images. Cells were detected based on their optical density and positivity (CD8^+^ or Ki67^+^) was assessed based on the amount of DAB signal in each cell. Results were expressed as a number of stained cells by square millimeter of analyzed tissue.

### Flow cytometry

The day after the last administration of MRTX1257 (= D4 after RT), animals were sacrificed by cervical dislocation and subcutaneous tumors were harvested. Tumors were mechanically and then enzymatically dissociated by using the “Tumor Dissociation Kit Mouse” (Miltenyi Biotec, Germany) for 30 min at 37 °C and 1750 rpm. Cell suspensions were then filtered using 70 µm cell strainers (Miltenyi Biotec, Germany), and further used for Fc receptor blockage and then immune-cell staining.

For Fc receptor blockage, cell suspensions were incubated with purified anti-mouse CD16/32 (clone 93; BioLegend) during 10 min at 4 °C. Subsequently, cell populations were stained 20 min at 4 °C using antibodies listed in supplementary table and at dilutions recommended by the manufacturer.

For tumor-infiltrating immune cell staining, anti-CD45 (REA737), anti-Ly6G (REA526), anti-CD11c (REA754; Miltenyi Biotec), anti-CD11b (M1/70; BD Horizon), anti-Ly6C (HK 1.4) and anti-CD64 (X54-5/7.1; BioLegend) were used to identify non-immune cells (CD45-), myeloid cells (CD45^+^ CD11b^+^), macrophages (CD45^+^ CD11b^+^ Ly6G^−^ Ly6C^−/low^ CD64^+^), inflammatory monocytes (CD45^+^ CD11b^+^ Ly6G^−^ Ly6C^high^ CD64^−^) and conventional dendritic cells type 2 (cDC2: CD45^+^ CD11b^+^ Ly6G^−^ CD64^−^ CD11c^+^). Anti-CD45 (REA737; Miltenyi Biotec), anti-CD8 (53–6.7; BD Horizon), anti-CD4 (RM 4–5), anti-CD11b (M1/70) and anti-NKp46 (29A 1.4; BioLegend) were used to identify lymphoid cells (CD45^+^ CD11b^−^) CD4^+^ T cells (CD45^+^, CD11b^−^ CD4^+^), CD8^+^ T cells (CD45^+^ CD11b^−^ CD8^+^), NK cells (CD45^+^ CD11b^−^ NKp46^+^). Furthermore, after fixation and permeabilization using the « Mouse FoxP3 Buffer Set» (BD Pharmingen), anti-FoxP3 (REA788; Miltenyi Biotec) was used to distinguish between conventional CD4^+^ T cells (CD45^+^ CD11b^−^ CD4^+^ FoxP3^−^) and Treg cells (CD45^+^ CD11b^−^ CD4^+^ FoxP3^+^).

Anti-PD1 (REA802), anti-CD80 (REA983), anti-MHC II (REA813; Miltenyi Biotec) and anti-PD-L1 (MIH5; BD Optibuild) were used. For these markers, mean fluorescence intensities (MFI) for each target population were normalized to an unstained control in each treatment group (DeltaMFI).

Samples were acquired on a LSR Fortessa X20 (BD, Franklin Lakes, NJ) with FACSDiva software, and data were analyzed using FlowJo V10.8.1 software (Tree Star, Ashland, OR).

### Statistical analysis

Statistical analysis was performed using GraphPad Prism Software version 8. Differences between groups were analyzed using a one-way ANOVA followed by Dunnett’s correction. All values were expressed as means ± standard errors to mean (SEM). All analyses were two-sided and a difference with a P-value < 0.05 was considered statistically significant. Regarding survival data, Kaplan–Meier curves were compared using the log-rank test, with P-values < 0.05 considered statistically significant.

## Results

### *MRTX1257 radio-sensitizes CT26 and LL2 *in vitro* depending on RAS mutation profile*

**Fig. 1:**
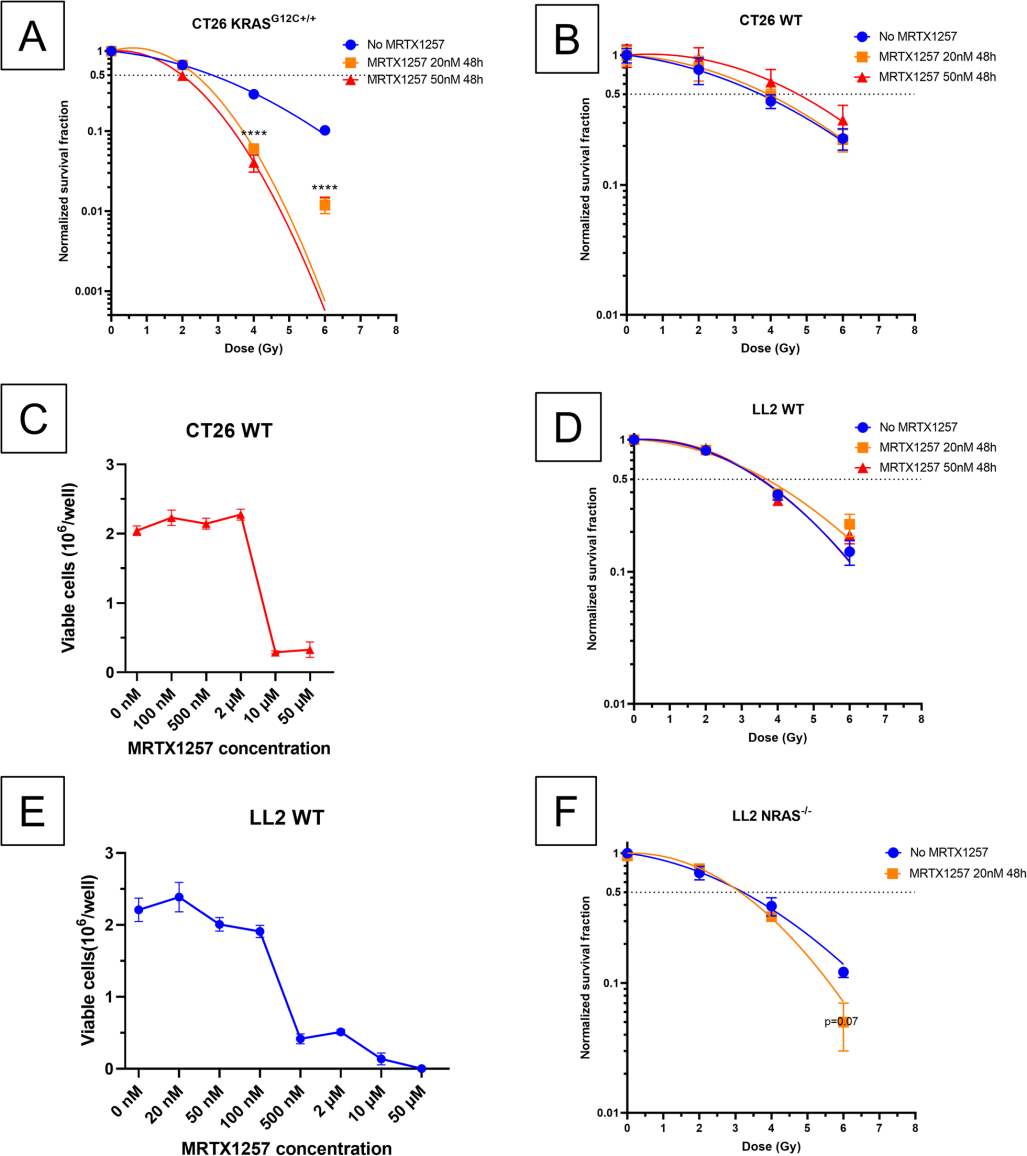
MRTX1257 radio-sensitizes in vitro CT26 and LL2 tumor cell lines according to their KRAS mutational status. Clonogenic survival assays were performed after treatment using RT and MRTX1257 at various doses in different cell lines harboring different KRAS mutational profiles. Normalized survival fractions are represented in mean ± standard-error to mean (SEM), with n = 3 to 6 replicates per condition. Survival curves are extrapolations according to the linear quadratic model. ****: p < 0.0001 (one-way ANOVA). Survival curves for (**A**) CT26 KRAS^G12C+/+^; **B** CT26 WT; **C** LL2 NRAS^−/−^; **D** LL2 WT. **E, F** Dose–response curves for CT26 WT and LL2 WT cell lines exposed to various concentrations of KRAS.^G12C^ inhibitor MRTX1257

We first assessed in vitro the efficacy of the combination between MRTX1257 at various concentrations and different RT doses in CT26 cell lines harboring different RAS mutation profiles. MRTX1257 was able to radio-sensitize CT26 KRAS^G12C+/+^ cells when used at the concentrations of 20 and 50 nM for 48 h after irradiation (normalized survival fractions at 4 Gy: 0.06 and 0.04 respectively at 20 nM and 50 nM versus 0.23 in control group; p < 0.0001) (Fig. 1A).

Regarding CT26 WT cells, which are reported in literature as harboring homozygous KRAS^G12D^ mutation [28, 29], MRTX1257 at 20 nM or 50 nM for 48 h did not increase their radio-sensitivity at the tested RT doses, i.e. from 2 to 6 Gy (Fig. 1B). To explore the absence of efficacy of MRTX1257 in this cell line, we performed a dose-escalation assay by using increasing concentrations of MRTX1257 in vitro. The results showed a half maximal inhibitory concentration (IC50) between 2 and 10 µM, far beyond the 20 to 50 nM used in CT26 KRAS^G12C+/+^ (Fig. 1C).

We then explored MRTX1257 in association with RT in two different LL2 cell lines. Regarding LL2 WT cells, we decided to sequence the gene *Kras* and found this cell line harbors *KRAS*^*G12C*±^ heterozygous mutation (data not shown). MRTX1257 at 20 or 50 nM for 48 h did not influence the radio-sensitivity of this cell line (Fig. 1D), which suggests that a heterozygous G12C mutation of *KRAS* is not sufficient to allow a proper efficacy of the combination treatment. As in CT26 WT cell line, we performed a dose-escalation assay in LL2 WT cell line and found an IC50 between 100 and 500 nM (Fig. 1E), which is inferior to the IC50 in CT26 WT cell line and may be explained by the presence of a single *KRAS*^*G12C*^ mutation.

LL2 NRAS^−/−^ cells harbor both a heterozygous mutation of *KRAS* and a knock-out (KO) mutation of *NRAS*. Although we observed a trend in radio-sensitizing these cells using MRTX1257 at 20 nM for 48 h, this effect was not statistically significant (p = 0.07) (Fig. 1F). This suggests that, even when silencing *NRAS*, a heterozygous KRAS^G12C^ mutation is not sufficient to provide a strong radio-sensitizing effect of MRTX1257 comparable to the one observed in KRAS^G12C+/+^ cells.

Of note, the use of MRTX1257 at the concentrations of 5 and 10 nM during 24 h did not provide any radio-sensitizing effect in both CT26 KRAS^G12C+/+^ and LL2 NRAS^−/−^ tumor cells (Additional file 1: Figure S1A, B

Overall, these data show MRTX1257 is able to radio-sensitize tumor cells in vitro depending on their *RAS* mutational profile. Moreover, the impact of *KRAS*^*G12C*^ mutational status on this effect was predominant, since *NRAS* mutational status had a moderate, non-significant impact on it in addition to *KRAS* status.

### ***MRTX1257 increases the efficacy of RT in nude mice bearing CT26 KRAS***^***G12C***+***/***+^***tumors***

After the demonstration of in vitro radio-sensitization of CT26 KRAS^G12C+/+^ using MRTX1257, we aimed to explore a similar setting of combinatorial approach in athymic nude mice bearing CT26 KRAS^G12C+/+^ tumors. This allowed us to assess the in vivo efficacy of the combination of MRTX1257 and RT in a lymphoid compartment-depleted setting, serving as an extension of the previous in vitro experiment.

**Fig. 2:**
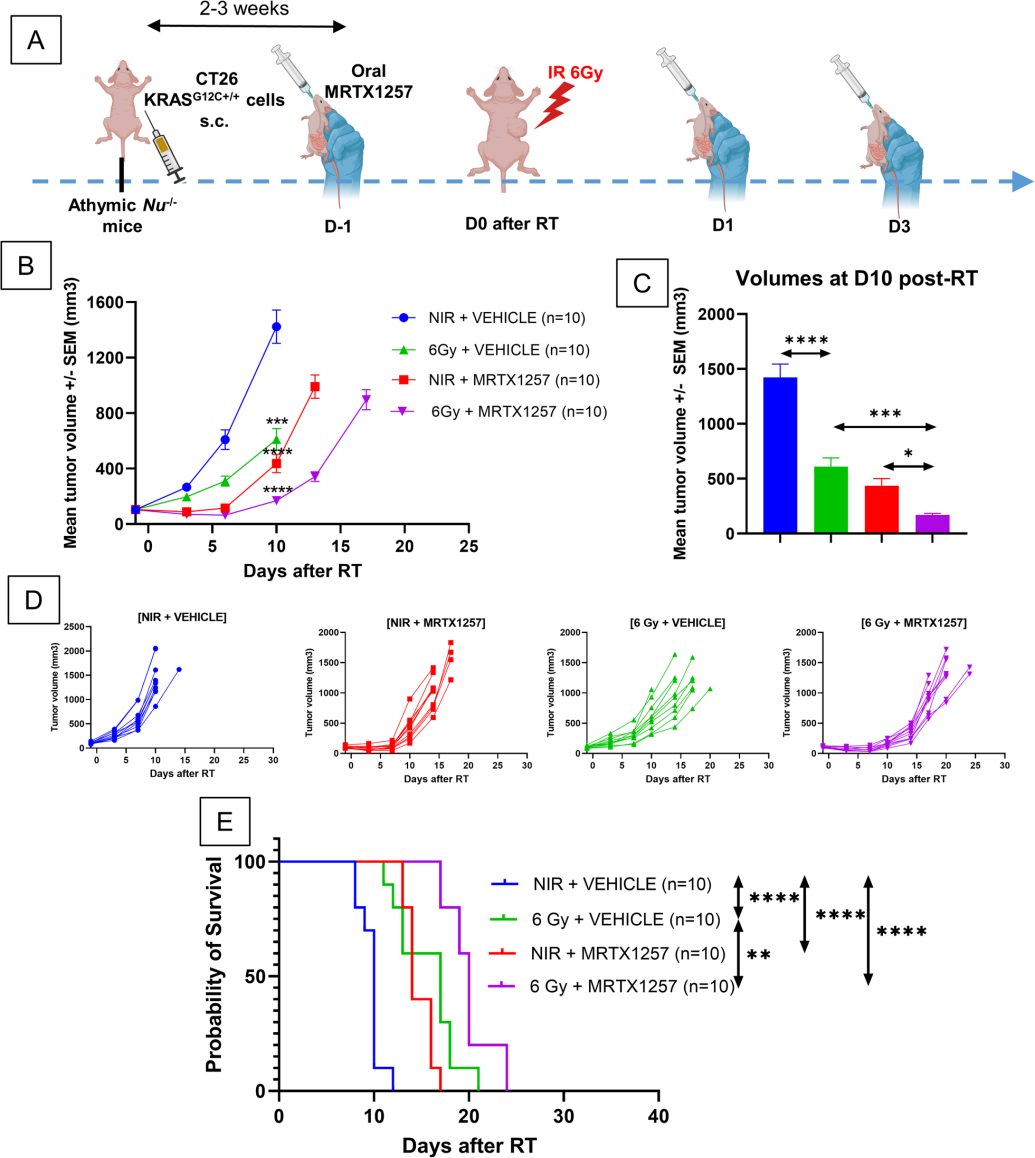
MRTX1257 increases the efficacy of RT in nude mice bearing CT26 KRAS^G12C+/+^ however without achieving durable responses **A** Athymic nude mice were subcutaneously inoculated with CT26 KRAS^G12C+/+^ cells. Once the tumors reached an average volume of 90–100 mm^3^, mice received via oral administration 50 mg/kg of MRTX1257 or vehicle. The day after, mice received a single fraction of 6 Gy on the tumor mass. MRTX1257 at the dose of 50 mg/kg or vehicle were then administered at D1 and D3 after RT. The experiment was repeated twice. **B** Tumor growth (mean ± SEM) ***: p < 0.001; ****: p < 0.0001 (two-way ANOVA). **C** Mean tumor volumes in each group 10 days after RT are represented. *: p < 0.05; ***: p < 0.001; ****: p < 0.0001 (one-way ANOVA). **D** Individual growth profiles of CT26 KRAS.^G12C+/+^ tumors are represented for each condition. **E** Survival Kaplan–Meier curves were compared between each group using the log-rank test. **: p < 0.01; ****: p < 0.0001

MRTX1257, administered three times at 50 mg/kg (Fig. 2A) increased the efficacy of a single-dose of 6 Gy delivered on CT26 KRAS^G12C+/+^ tumors in nude mice. Regarding the tumor growth curve (Fig. 2B) as well as the average tumor volume in each group at D10 after radiotherapy (Fig. 2C), the differences are significant in all treatment groups compared to control group (p < 0.0001). Moreover, the group treated with combination treatment shows better outcomes compared to both the groups treated with irradiation alone (p = 0.005) and with MRTX1257 alone (p = 0.03).

This translated in a better survival in the group treated with combination (p < 0.0001 versus the control group and MRTX1257 alone group, p = 0.006 versus the irradiation alone group) (Fig. 2D and 2E). However, despite these interesting results, none of the conditions tested was able to induce a durable response in nude mice, since all mice finally relapsed. This observation may be due to the putative involvement of a functional and complete immune compartment in achieving durable responses following RT and MRTX1257.

### ***MRTX1257 increases the efficacy of RT in immunocompetent BALB/c mice bearing CT26 KRAS***^***G12C***+***/***+^***tumors, and is able to induce durable responses in combination with RT***

We then decided to explore the efficacy of the combination of MRTX1257 and RT in immunocompetent BALB/c mice bearing CT26 KRAS^G12C+/+^ tumors.

**Fig. 3:**
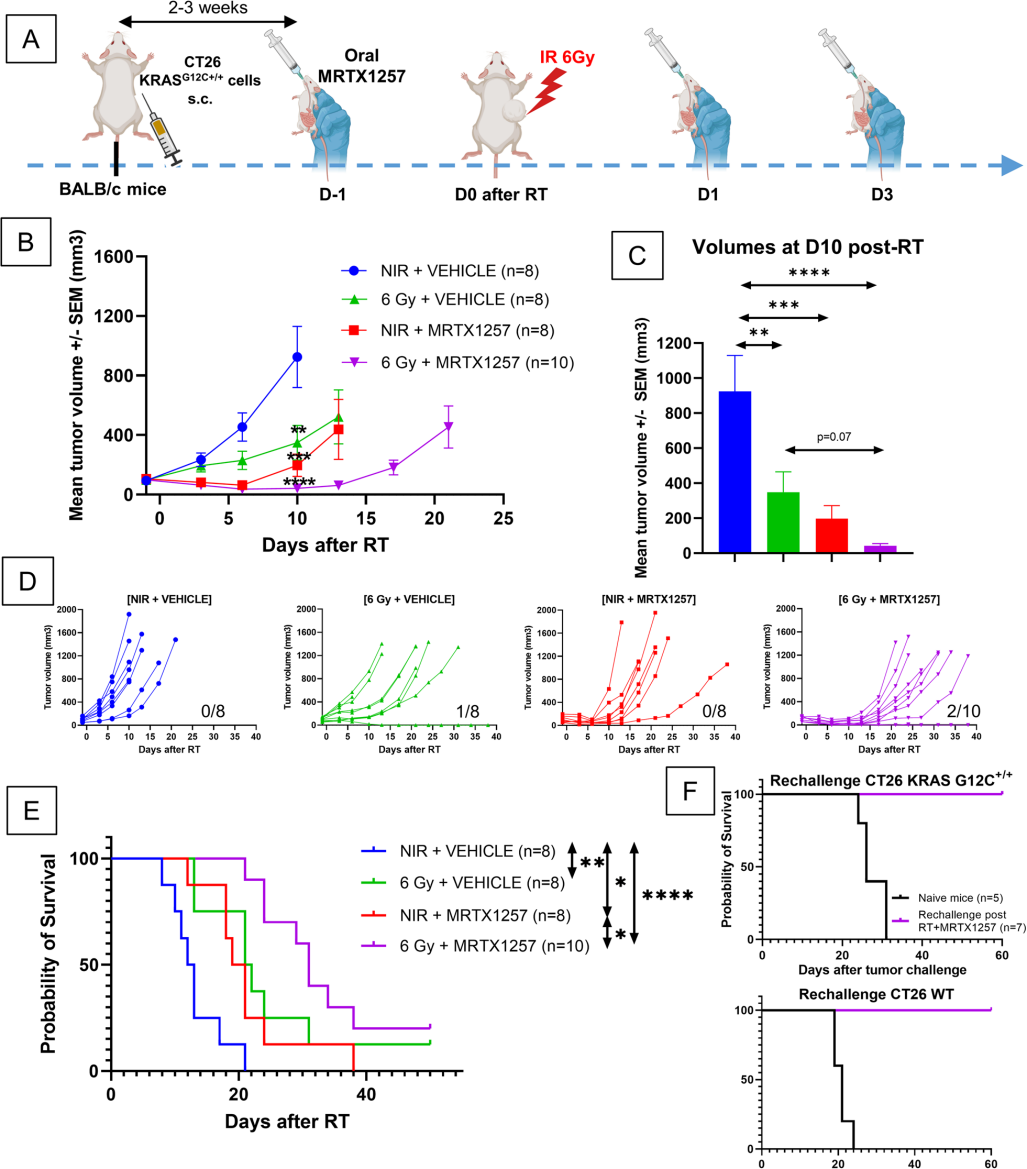
MRTX1257 increases the efficacy of RT in immunocompetent BALB/c mice bearing CT26 KRAS^G12C+/+^ tumors and achieved durable responses in association with RT. **A** BALB/c mice were subcutaneously inoculated with CT26 KRAS^G12C+/+^ cells. Once the tumors reached an average volume of 90–100 mm^3^, mice received via oral administration 50 mg/kg of MRTX1257 or vehicle. The day after, mice received a single fraction of 6 Gy on the tumor mass. MRTX1257 at the dose of 50 mg/kg or vehicle were then administered at D1 and D3 after RT. The experiment was repeated twice. **B** Tumor growth (mean ± SEM). **: p < 0.01; ***: p < 0.001; ****: p < 0.0001 (two-way ANOVA). (**C**) Mean tumor volumes in each group 10 days after RT are represented. **: p < 0.01; ***: p < 0.001; ****: p < 0.0001 (one-way ANOVA). **D** Individual growth profiles of CT26 KRAS G12C^+/+^ tumors are represented for each condition. The number of mice achieving durable response with no tumor being assessed at the end of the experiment is indicated below each panel. **E** Survival Kaplan–Meier curves were compared between each group using the log-rank test. *: p < 0.05; **: p < 0.01; ****: p < 0.0001. **F** Immunocompetent BALB/c mice bearing CT26 KRAS^G12C+/+^ tumors and cured by the combination of RT and MRTX1257 were subsequently rechallenged with either 1.10^6^ CT26 KRAS^G12C+/+^ (top) or 5.10^5^ CT26 WT cells (bottom) subcutaneously injected in their contralateral flank. Their survivals were then compared with those of naive mice receiving similar injections

As in nude mice, MRTX1257 administered 3 times at 50 mg/kg (Fig. 3A) increased the efficacy of RT delivered at a single dose of 6 Gy. Indeed, the tumor growth rate decreased in all treated groups compared with the control untreated group with a maximum of efficacy reached in the combination group (irradiation alone: p = 0.004; MRTX1257 alone: p = 0.0005; irradiation + MRTX1257: p < 0.0001) (Fig. 3B). Moreover, the difference in average tumor volume at D10 between the irradiation alone group and the combination group, even if not meeting the statistical significance threshold, was notable (p = 0.07, Fig. 3C). Furthermore, as illustrated in the individual growth profiles (Fig. 3D) and in the survival curves (Fig. 3E), mice in the group treated with MRTX1257 alone relapsed quickly after the end of the treatment whereas those in the combination group showed a better survival (p = 0.04), with 2 mice out of 10 achieving durable responses. To determine if the combination treatment using irradiation and MRTX1257 led to significant anti-tumor immune memory in mice, we rechallenged the mice showing complete response with s.c. injection of CT26 KRAS^G12C+/+^ or CT26 WT cells in contralateral flank. None of the rechallenged mice developed new tumors, in contrast with 100% of naive mice receiving similar s.c. injections, suggesting a potent anti-tumor immune memory provided by the combination treatment against both CT26 KRAS^G12C+/+^ and CT26 WT tumor cells (Fig. 3F).

Of note, no significant toxicities were observed in mice when treating with MRTX1257 and RT in these conditions.

As a reminder, we did not observe any durable response in nude mice and, as illustrated in their individual growth profiles (Fig. 2D), all nude mice experienced fast and homogeneous relapses. This suggests that the presence of a functional lymphoid compartment has an impact on the efficacy of the combination of RT and MRTX1257.

We then explored whether the combination of irradiation and MRTX1257 was able to generate systemic anti-tumor immune response leading to the abscopal response of a lesion outside of the irradiation field. To this end, we administered this combination in BALB/c mice bearing 2 s.c. CT26 KRAS^G12C+/+^ tumors of which only one received irradiation (Additional file 1: Fig. S2A). In primary (irradiated) tumors, in a comparable manner than in the one-tumor experiments described above, MRTX1257 increased the anti-tumor efficacy of RT ([RT + MRTX1257] VS RT alone: p = 0.003). Moreover, at late timepoint (D13 after RT), the combined treatment showed an interesting anti-tumor superiority compared to MRTX1257 alone, although not reaching the significance criteria due to the low number of mice included in each group (p = 0.10) (Additional file 1: Figure S2B). In secondary (unirradiated) tumors, the addition of RT to the primary tumor failed to improve the efficacy of MRTX1257, thus suggesting the absence of abscopal effect in this setting (Additional file 1: Figure S2C). These findings translated into the absence of significant benefit of survival in mice treated with primary tumor irradiation and MRTX1257 compared to those treated with MRTX1257 only (Additional file 1: Figure S2D).

### MRTX1257 does not show any significant efficacy in CT26 WT derived tumors

**Fig. 4:**
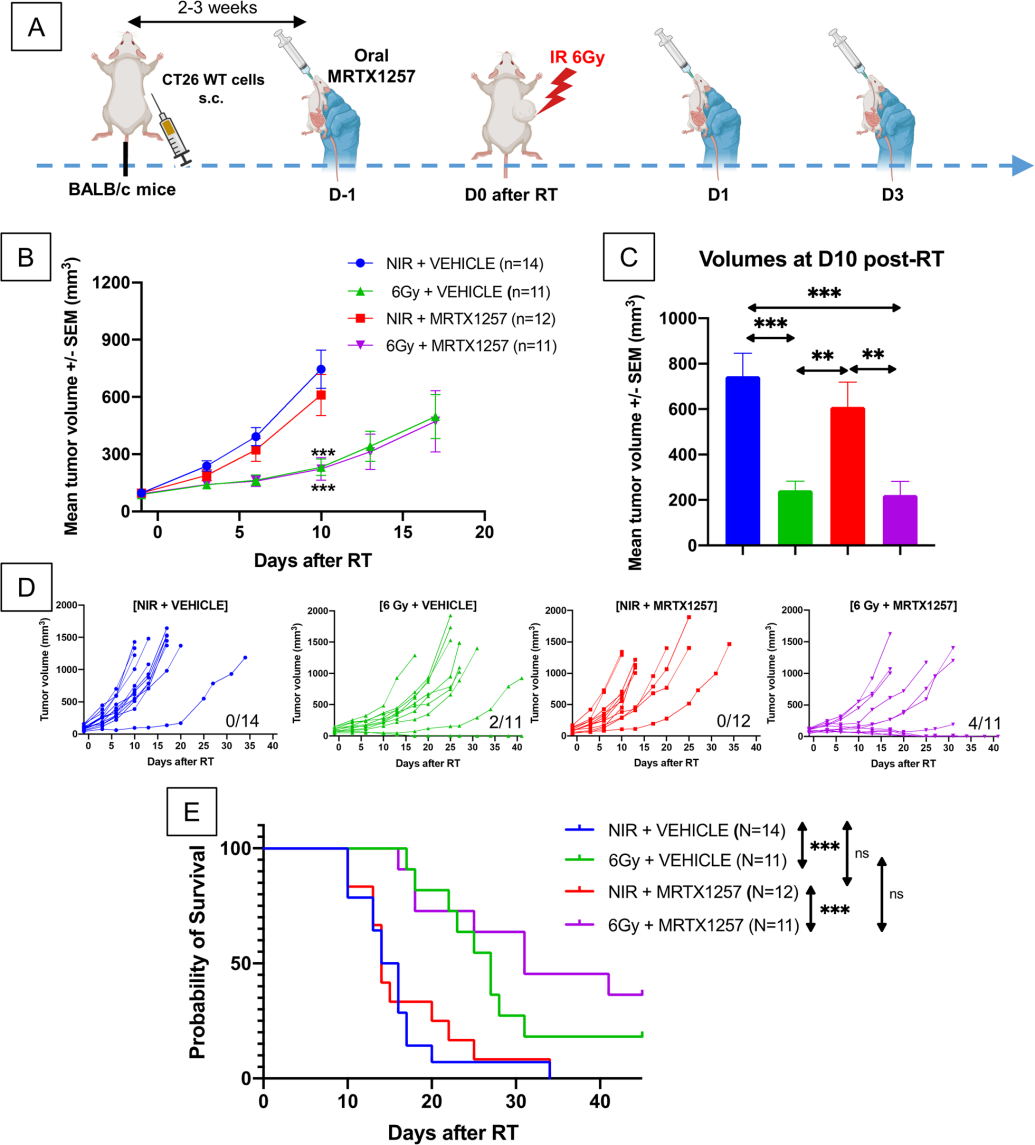
MRTX1257 does not have any significant effect alone or in combination with RT in immunocompetent BALB/c mice bearing CT26 WT tumors. **A** BALB/c mice were subcutaneously inoculated with CT26 WT cells. Once the tumors reached an average volume of 90–100 mm^3^, mice received via oral administration 50 mg/kg of MRTX1257 or vehicle. The day after, mice received a single fraction of 6 Gy on the tumor mass. MRTX1257 at the dose of 50 mg/kg or vehicle were then administered at D1 and D3 after RT. The experiment was repeated three times. **B** Tumor growth (mean ± SEM). ***: p < 0.001 (two-way ANOVA). **C** Mean tumor volumes in each group 10 days after RT are represented. **: p < 0.01; ***: p < 0.001 (one-way ANOVA). **D** Individual growth profiles of CT26 KRAS G12C.^+/+^ tumors are represented for each condition. The number of mice achieving durable response with no tumor being assessed at the end of the experiment is indicated below each panel. **E** Survival Kaplan–Meier curves were compared between each group using the log-rank test. ***: p < 0.001

To determine if the efficacy of the combination between RT and MRTX1257 relies upon the presence of *KRAS*^*G12C*^ mutation in CT26 tumors, we performed a similar tumor growth experiment in BALB/c mice bearing CT26 WT tumors (Fig. 4A).

A significant decrease in tumor growth rate was observed in both groups treated using RT compared to the control group (p = 0.0006 for groups treated with RT alone and RT + MRTX1257) (Fig. 4B and C). However, we did not observe any positive effect on tumor growth rate attributable to the administration of MRTX1257 alone or in combination with RT in mice bearing CT26 WT tumors.

Regarding the survival outcomes, survival was improved only in groups treated with RT compared to their non-irradiated counterparts [(6 Gy + VEHICLE) versus (NIR + VEHICLE): p = 0.001; (6 Gy + MRTX1257) versus (NIR + MRTX1257): p = 0.0009]. Interestingly, we were able to cure 4 mice out of 11 in the combination group, which is higher than in CT26 KRAS^G12C+/+^ tumors. However, we were also able to cure 2 mice out of 11 in the irradiation alone group, with similar associated tumor growth profiles (Fig. 4D) and non-statistically different survival (Fig. 4E). Taken together, these data suggest a higher level of radio-sensitivity of CT26 WT tumors compared to CT26 KRAS^G12C+/+^.

### ***MRTX1257 increases the anti-proliferative effects of RT in CT26 KRAS***^***G12C***+***/***+^***tumors but not in CT26 WT tumors***

**Fig. 5:**
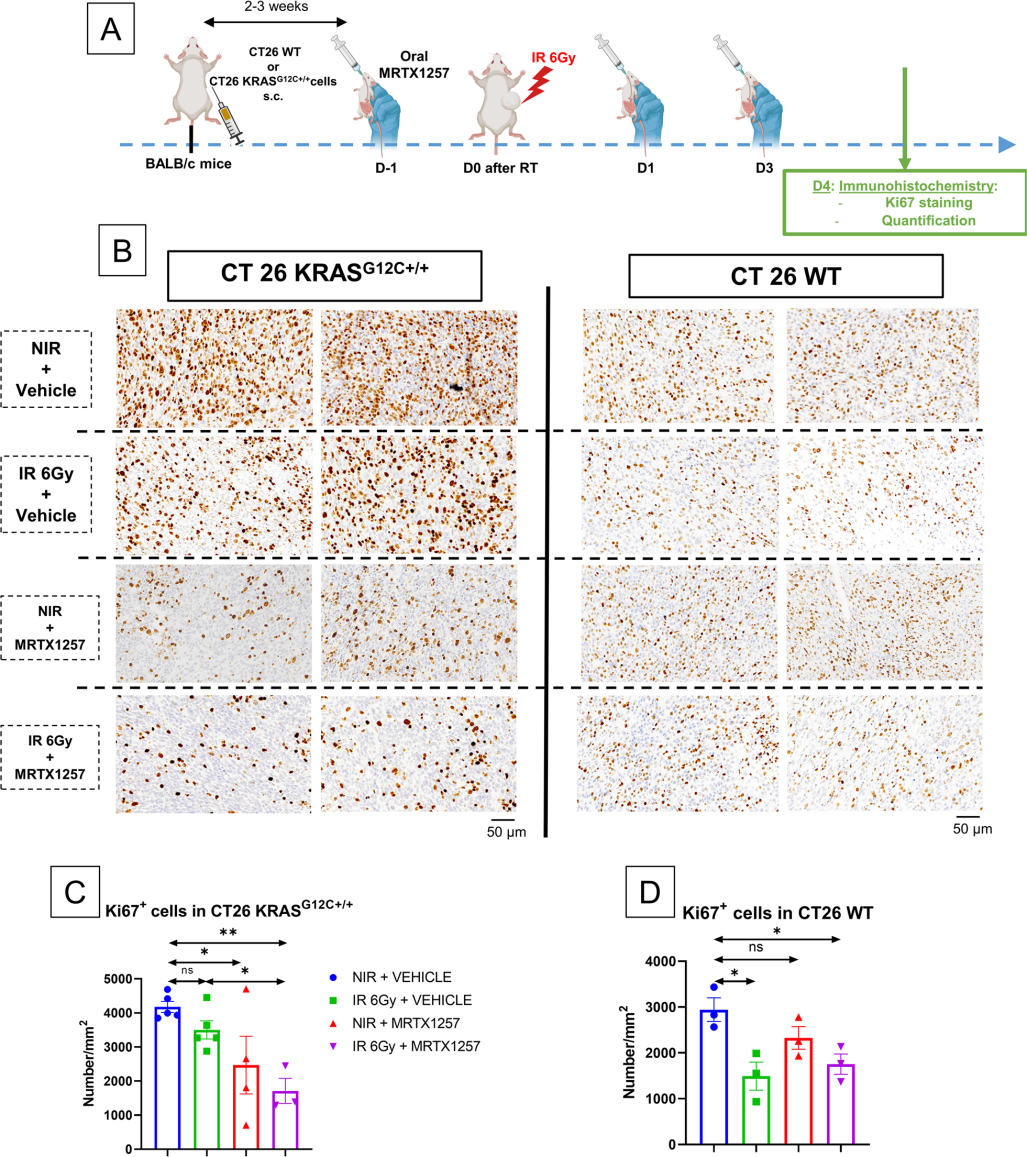
MRTX1257 increases the anti-proliferative effect of RT in CT26 KRAS^G12C+/+^ tumors but not in CT26 WT tumors. **A** BALB/c mice were subcutaneously inoculated with CT26 WT cells or CT26 KRAS^G12C+/+^ cells. Once the tumors reached an average volume of 90–100 mm^3^, mice received via oral administration 50 mg/kg of MRTX1257 or vehicle. The day after, mice received a single fraction of 6 Gy on the tumor mass. MRTX1257 at the dose of 50 mg/kg or vehicle were then administered at D1 and D3 after RT. At D4 after RT, mice were sacrificed, tumors were harvested and then fixated using paraformaldehyde (PFA) 4%. Immunohistochemistry for Ki67 and then the quantification of Ki67^+^ cells were performed. **B** Photographs at magnification 13X of 4 µm-thick CT26 KRAS^G12C+/+^ (left) or CT26 WT (right) tumor slices stained with anti-Ki67 antibody. Two representative photographs, each of them from a different tumor, are represented for each condition. **C** Quantification of Ki67^+^ cells per mm^2^ in CT26 KRAS G12C^+/+^ tumor slices. Each point is representative of a single tumor. Mean ± standard-errors to mean (SEM). N = 3–5 tumors/group. *: p < 0.05; **: p < 0.01 (one-way ANOVA). **D** Quantification of Ki67^+^ cells per mm.^2^ in CT26 WT tumor slices. Each point is representative of a single tumor. Mean ± standard-errors to mean (SEM). n = 3 tumors/group. *: p < 0.05 (one-way ANOVA)

To assess the anti-proliferative effect of the association between RT and MRTX1257 in CT26 KRAS^G12C+/+^ and CT26 WT tumors, we performed immunohistochemistry (IHC) analyses in both tumor types the day after the last administration of MRTX1257, i.e. D4 after RT (Fig. 5A).

Although irradiation alone was not able to significantly decrease the expression of Ki67 within CT26 KRAS^G12C+/+^ tumors, the combination of RT with MRTX1257 greatly decreased the density of Ki67^+^ cells within these tumors (p = 0.003 versus the control) (Fig. 5B, C). Moreover, in CT26 KRAS^G12C+/+^ tumors, the combined treatment significantly decreased the density of Ki67^+^ cells compared to irradiation alone (p = 0.02), thus confirming the ability of MRTX1257 to increase the efficacy of RT in CT26 KRAS^G12C+/+^ tumors, in line with the tumor growth experiments.

In CT26 WT tumors, unlike in CT26 KRAS^G12C+/+^ tumors, the expression of Ki67 decreased only in irradiated tumors, with or without the adjunction of MRTX1257 (p = 0.02 and p = 0.03 respectively for irradiation alone versus the control and combination versus the control) (Fig. 5B, D). This is in line with the tumor growth experiments showing the absence of efficacy of MRTX1257 in CT26 WT tumors.

### ***CD8***^+^***T cells are not sufficient to explain the efficacy of the association between RT and MRTX1257 in CT26 KRAS***^***G12C***+***/***+^***tumors***

**Fig. 6:**
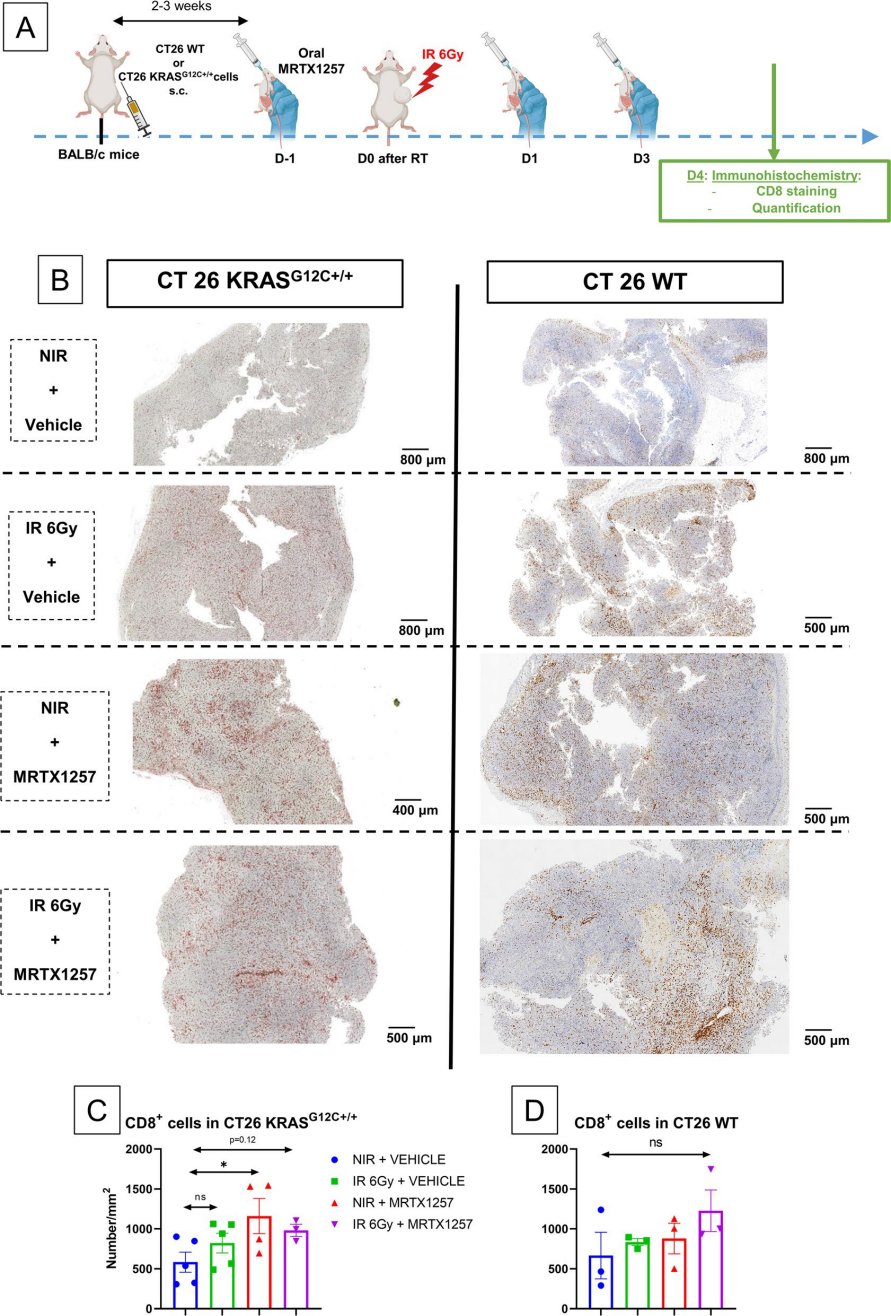
An increase in tumor-infiltrating CD8^+^ T cells is not sufficient to explain the radio-sensitizing effect of MRTX1257. **A** BALB/c mice were subcutaneously inoculated with CT26 WT cells or CT26 KRAS G12C^+/+^ cells. Once the tumors reached an average volume of 90–100 mm^3^, mice received via oral administration 50 mg/kg of MRTX1257 or vehicle. The day after, mice received a single fraction of 6 Gy on the tumor mass. MRTX1257 at the dose of 50 mg/kg or vehicle were then administered at D1 and D3 after RT. At D4 after RT, mice were sacrificed, tumors were harvested and then fixated using paraformaldehyde (PFA) 4%. Immunohistochemistry analyses for CD8 and then the quantification of CD8^+^ cells were performed. **B** Photographs at magnification 1.5X of 4 µm-thick CT26 KRAS^G12C+/+^ (left) or CT26 WT (right) tumor slices stained with anti-CD8 antibody. **C** Quantification of CD8^+^ cells per mm^2^ in CT26 KRAS^G12C+/+^ tumor slices. Each point is representative of a single tumor. Mean ± standard-errors to mean (SEM). n = 3–5 tumors/group. *: p < 0.05 (one-way ANOVA). **D** Quantification of CD8^+^ cells per mm^2^ in CT26 WT tumor slices. Each point is representative of a single tumor. Mean ± standard-errors to mean (SEM). n = 3 tumors/group. Ns: non-significant (one-way ANOVA)

We then performed the staining and the quantification of CD8^+^ cells within both CT26 KRAS G12C^+/+^ and CT26 WT tumors (Fig. 6A).

Compared to the control group, the density of CD8^+^ cells within CT26 KRAS^G12C+/+^ tumors was increased in the group treated using MRTX1257 alone (p = 0.05), but not in the combined treatment group (p = 0.12) (Fig. 6B, C). This result shows that the radio-sensitizing effect induced by MRTX1257 in CT26 KRAS^G12C+/+^ tumors cannot be solely attributed to an increase in the infiltration of CD8^+^ T cells within the microenvironment of these tumors, which is consistent with the efficacy of the combined treatment also observed in T cell-deficient nude mice.

Regarding CT26 WT tumors, we did not observe any significant difference in the density of CD8^+^ cells within tumors regardless of the treatment (Fig. 6B, D).

### ***In CT26 KRAS***^***G12C***+***/***+^***tumors, MRTX1257 drives the down-regulation of PD-L1 and counteracts its upregulation following RT alone***

**Fig. 7:**
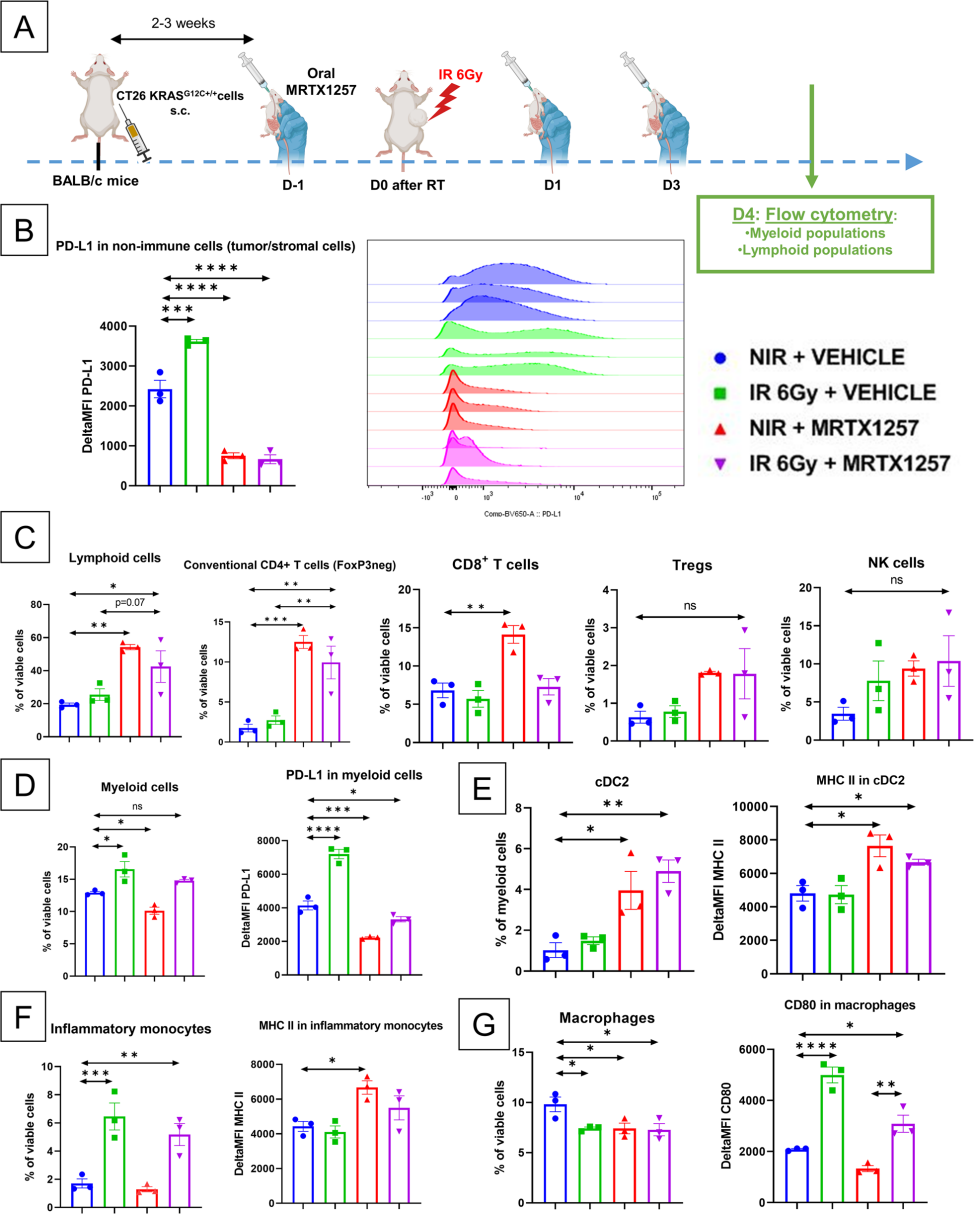
RT and MRTX1257 reshape the tumor immune microenvironment in CT26 KRAS^G12C+/+^ tumors. Flow cytometry analyses were performed in single-suspensions derived from CT26 KRAS^G12C+/+^ tumors. Results are represented in mean ± standard-error to mean (SEM). Each point represents a single tumor in both the untreated control group (NIR + VEHICLE) and the group treated with RT alone (IR 6 Gy + VEHICLE). Each point represents 2 different tumors pooled in a single sample in both the group treated with MRTX1257 alone (NIR + MRTX1257) and the group treated with combined RT and MRTX1257 (IR 6 Gy + MRTX1257). n = 3/group. Except for dendritic cells, the proportions of the different immune cell subtypes are expressed in percentage in viable cells whereas the expression of PD-L1, major histocompatibility complex II (MHC II), and CD80 are expressed in the difference in mean fluorescence intensity compared to an unstained control within each condition (Delta MFI). NS: non-significant; *: p < 0.05; **: p < 0.01; ***: p < 0.001; ****: p < 0.0001 (one-way ANOVA) **A** BALB/c mice were subcutaneously inoculated with CT26 KRAS^G12C+/+^ cells. Once the tumors reached an average volume of 90–100 mm^3^, mice received via oral administration 50 mg/kg of MRTX1257 or vehicle. The day after, mice received a single fraction of 6 Gy on the tumor mass. MRTX1257 at the dose of 50 mg/kg or vehicle were then administered at D1 and D3 after RT. At D4 after RT, mice were sacrificed, tumors were harvested and were used to perform flow cytometry. **B** Expression of PD-L1 in tumor and stromal CD45^−^ cells. Mean ± SEM (left panel) and histogram overlay (right panel). **C** Proportion of lymphoid cells, conventional CD4 + T cells, CD8 + T cells and Tregs. **D** Proportion of myeloid cells and expression of PD-L1 within myeloid cells. **E** Proportion of dendritic cells type 2 (cDC2) in percentage of myeloid cells and expression of MHC II within cDC2. **F** Proportion of inflammatory monocytes and expression of MHC II within inflammatory monocytes. **G** Proportion of macrophages and expression of CD80 within macrophages

The tumor growth experiments exposed earlier in this article showed that, after treatment, nude mice bearing CT26 KRAS^G12C+/+^ relapsed faster than immunocompetent BALB/c mice. Thus, we did not observe any durable response in nude mice treated with the combination. Therefore, we hypothesized MRTX1257 could reshape the immune microenvironment of CT26 KRAS^G12C+/+^ tumors into a pro-inflammatory and anti-tumor phenotype that contributes to the radio-sensitizing effect observed in these tumors. We hence used flow cytometry to explore the microenvironment of CT26 KRAS^G12C+/+^ tumors harvested from BALB/c mice (Fig. 7A).

First, in tumor and stromal cells, identified as CD45 negative cells, we found the expression of PD-L1 was upregulated following RT alone (p = 0.005) while it was dramatically downregulated in both MRTX1257 alone and combination groups (p < 0.0001) (Fig. 7B). The same outcomes were observed in myeloid cells, with the upregulation of PD-L1 following RT alone (p < 0.0001) and its downregulation in groups treated with MRTX1257 alone (p = 0.0003) or the combined treatment (p = 0.02) (Fig. 7D).

Then, the proportion of lymphoid cells was increased in groups treated with MRTX1257 alone and the combination compared to the control group. Among the lymphoid subtypes, the proportion of conventional CD4^+^ T cells, defined as FoxP3 negative CD4^+^ T cells, was increased in both the groups treated with MRTX1257 alone and with the combined treatment. (Fig. 7C).

However, regarding the proportion of CD8^+^ T cells, we did not observe any difference between the irradiation alone group and the combination group, while this proportion increased in the MRTX1257 alone group (MRTX1257 alone versus control: p = 0.004). This is in line with the quantification of CD8^+^ cells in the IHC experiment above. In contrast, we observed a non-significant trend in the increase of NK cells following the combination treatment compared to the untreated control condition (p = 0.14) (Fig. 7C).

The analysis of the myeloid compartment also revealed the microenvironment of CT26 KRAS^G12C+/+^ tumors was reshaped depending on the treatment condition. Indeed, the combination treatment increased the proportion of conventional dendritic cells type 2 (cDC2) and their expression of MHC class II within the tumor immune microenvironment (Fig. 7E). Moreover, the combined treatment increased the proportion of inflammatory monocytes within the tumor microenvironment (p = 0.008) while it decreased the proportion of macrophages (p = 0.02). However, RT and the combined treatment were able to polarize macrophages into a pro-inflammatory and anti-tumor phenotype characterized by the upregulation of the activation marker CD80 (Fig. 7F, G).

Taken together, these results highlight the different impacts of RT and MRTX1257 on the lymphoid and the myeloid compartments within CT26 KRAS^G12C+/+^ tumors, with therefore the combinatorial approach taking benefit of the positive effects of both treatments. They also highlight an important decrease in the expression of PD-L1 within tumor cells and myeloid cells following MRTX1257 alone and RT + MRTX1257, which is likely to reshape the tumor immune microenvironment into a pro-inflammatory, anti-tumor phenotype, contributing to the efficacy of the combination.

## Discussion

To our knowledge, this study is the first to explore the efficacy and immunological properties of the specific association of a KRAS^G12C^ inhibitor with RT in a preclinical animal model of KRAS^G12C^ mutated cancer. The recent work by Zheng et al. mainly focused on a triple association of RT, MEK inhibitor and KRAS inhibitor (AMG510), with therefore the difficulty to identify which features are attributable to each of these 3 treatments [30]. Moreover, we chose to perform flow cytometry directly in single-cell suspensions from CT26 *KRAS* mutated tumors, which is more reliable than a flow cytometry analysis of the spleen. In their study, RT in association with AMG510 was able to decrease the tumor growth of LLC tumors. This is in line with our results and confirms in vivo the radio-sensitizing effect of a potent KRAS inhibitor in preclinical setting, and therefore the interest to move towards the implementation of combinatorial strategies involving RT and KRAS inhibitors in KRAS-mutated cancers.

Furthermore, our results demonstrate the concomitant administration of MRTX1257 in multiple doses provides optimal efficacy and durable responses in CT26 KRAS^G12C+/+^ tumors in combination with RT. As a preliminary experiment (data not shown), we administered MRTX1257 in a single dose of 75 mg/kg in BALB/c mice bearing CT26 KRAS^G12C+/+^ tumors the day before RT. This resulted in the absence of efficacy of MRTX1257, and the only positive effects observed were due to RT, similarly to what we observed in CT26 WT tumors. Therefore, we next decided to administer MRTX1257 in 3 doses of 50 mg/kg at different time points both before and after RT. This resulted in an increase in the efficacy of RT due to MRTX1257 leading to the achievement of durable responses in immunocompetent mice and the generation of a potent anti-tumor immune memory against both CT26 KRAS^G12C+/+^ and CT26 WT cells. Such cross-reactive immune memory may result from the high clonal proximity between these two cell lines. Of note, in our study, we did not achieve any complete remission using MRTX1257 alone.

Moreover, MRTX1257 did not provide any effect in CT26 WT cell lines nor tumors, demonstrating the absence of off-target effect and therefore the safety of MRTX1257, which is a crucial issue in the particular setting of a combination with RT. This is in line with the absence of toxicity observed in mice treated with 3 administrations of 50 mg/kg of MRTX1257 alone or with RT.

Regarding the radio-sensitizing effect of MRTX1257 in CT26 KRAS^G12C+/+^ tumors, sensitizing *KRAS* mutant cells to radiation using KRAS inhibitors is a well-known strategy and various agents have been used in such a goal, of which prenyltransferase inhibitors [18], farnesyltransferase inhibitors [16], or antisense vectors [20]. However, these studies did not explore the immunological aspects of the association of RT and KRAS inhibitors in KRAS mutant tumors. In our study, the achievement of cures in 20% of the immunocompetent mice treated with the combination, in contrast with the fast relapses observed in nude mice, suggest the involvement of the immune compartment in the efficacy of the combined treatment. However, MRTX1257, alone or in combination with RT, significantly delayed tumor growth also in nude mice. Therefore, T cells, although crucial in the anti-tumor immune response, cannot be considered as the key pillar of the immunological outcomes observed following the combination. This deduction is in line with our results of IHC and flow cytometry not showing any significant increase in the infiltration of CD8^+^ T cells following the combined treatment.

In addition to CD8^+^ T cells, the proportion of NK cells within CT26 KRAS^G12C+/+^ tumors only showed a slight and non-significant increase following the combination of RT and MRTX1257. Overall, these results are in favor of the participation of non-lymphoid cell subtypes in the efficacy of the combined treatment. In line with such a hypothesis, our flow cytometry experiments in CT26 KRAS^G12C+/+^ tumors showed meaningful changes within the non-lymphoid compartment, including the downregulation of PD-L1 in myeloid cells as in tumor and stromal cells. The other meaningful changes observed in myeloid cells following the combination were an increase in the proportion of inflammatory monocytes and conventional dendritic cells type 2, as well as the upregulation of the activation marker CD80 within macrophages which is in favor of a more pro-inflammatory phenotype of these cells following RT alone or RT with MRTX1257.

The down-regulation of PD-L1 is a major positive effect of MRTX1257, and may be crucial for the efficacy of the combination as it counterbalances the upregulation of PD-L1 following RT, demonstrated in our study as in many other models [31, 32]. Moreover, the specific downregulation of PD-L1 in myeloid cells in the groups treated using MRTX1257 may be of major importance. Indeed, Strauss et al. demonstrated the specific ablation of PD-1 in myeloid cells more effectively decreased tumor growth compared to T cell-specific PD-1 ablation, notably by preventing the accumulation of myeloid-derived suppressor cells (MDSC) [33]. Therefore, this downregulation may contribute to the efficacy of RT + MRTX1257 in nude mice, but is not sufficient to induce long-term responses among them.

Overall, our immunological outcomes are in phase with most of those presented in the work by Briere et al. on MRTX849 used alone in preclinical models of KRAS^G12C^ mutated cancers, including an increase in the proportion of CD4^+^ T cells, CD4^+^ helper T cells and CD8^+^ T cells [26]. Moreover, our results are also congruent with the outcomes highlighted by Canon et al. with AMG510, including an increased infiltration of T cells and dendritic cells [34]. However, we are not able to compare the immunological outcomes for the association of RT with MRTX1257, since none of these studies associated KRAS inhibition with RT. It is notable that, in our immune profiling of CT26 KRAS^G12C+/+^ tumors, the lymphoid compartment appeared to be enhanced in the conditions treated with MRTX1257 whereas the myeloid compartment appeared to be enhanced following RT. A key feature of the combination of RT and MRTX1257 may be to leverage the enhancement of both of these immune cell compartments, a condition achievable only in immunocompetent mice. Therefore, one of the challenges for the future of combinations of RT with KRAS inhibitors will consist in identifying the individual impact of each immune subtype apart from the others and therefore benchmark the best strategies of combinations treatments in solid tumors harboring *KRAS*^*G12C*^ mutation.

Finally, recent data support a heterogeneity in the innate cancer cell radio-sensitivity itself, independent of cell cycle and microenvironment [35]. Taken together with a varying degree of dependence of cancer cells on the presence of *KRAS*^*G12C*^ mutations for growth and survival, as well as the heterogeneity of intratumoral *KRAS* mutation expression, our data highlight the importance of combination strategies in overcoming treatment resistance. This hypothesis is confirmed by the potent immune memory generated in mice bearing CT26 KRAS^G12C+/+^ tumors which have been cured following RT combined with MRTX1257. Indeed, this immune memory rejected both CT26 KRAS^G12C+/+^ and CT26 WT tumors subsequently implanted. In this complex setting, where monotherapies draw the limits of durable response achievement and most oncogene driven cancers relapse, while immune checkpoints benefit only a small patient group, defining new rational combinations is paramount.

## Conclusion

In this work, we first demonstrated the ability of MRTX1257, a potent covalent KRAS^G12C^ inhibitor analogous to MRTX849, to enhance the effect of radiotherapy both in vitro and in vivo. This effect depended on *RAS* mutational status, dose and timing of administration and was associated with a good safety profile. Moreover, the use of RT and MRTX1257 led to a significant cure rate in BALB/c mice bearing CT26 KRAS^G12C+/+^ tumors, but not in nude mice, highlighting the role of the tumor immune microenvironment in the radio-sensitizing effect of MRTX1257. This work constitutes a first step towards the implementation of new combinatorial approaches involving RT and MRTX1257 in KRAS G12C mutated cancers, with the aim of providing new therapeutic strategies with a prolonged clinical benefit. The optimal treatment sequencing and selected patient populations warrant further characterization both in the preclinical and clinical settings.

### Supplementary Information

**Additional file 1: Table S1.** List of the antibodies used and their respective dilutions in flow cytometry experiments. **Figure S1.** (complementary to Fig. 1): MRTX1257 at the concentration of 5 nM or 10 nM for 24 h does not sensitize CT26 KRAS^G12C+/+^ or LL2 NRAS^−/−^ tumor cells to radiation. Clonogenic survival assays were performed in CT26 KRAS^G12C+/+^ or LL2 NRAS^−/−^ tumor cells exposed to various concentrations of MRTX1257 for 24 h. Normalized survival fractions are represented in mean ± standard-error to mean (SEM), with n = 3 to 6 replicates per condition. Survival curves are extrapolations according to the linear quadratic model. Survival curves for **A** CT26 KRAS^G12C+/+^ cell line and **B** LL2 NRAS^−/−^ cell line. **Figure S2.** A single-fraction irradiation of 6 Gy does not increase the efficacy of MRTX1257 in a distant unirradiated tumor. The combination of RT delivered to a single-tumor and oral administration of MRTX1257 was experimented in BALB/c mice bearing bilateral s.c. CT26 KRAS^G12C+/+^ tumors according to the supplementary material and methods. **A** Schematic view of the experimental setting. A single fraction of 6 Gy was delivered to the right (primary) tumor (primary) whereas the left (secondary) tumor remained unirradiated. **B** Primary tumor volumes in each condition at the different timepoints (left), and specifically at D6 and D13 after RT (right). All the volumes are represented in mean ± standard-error to mean (SEM) (mm^3^). *: p < 0.05; **: p < 0.01; ****: p < 0.0001 (one-way ANOVA). **C** Secondary tumor volumes in each condition at the different timepoints (left), and specifically at D6 and D13 after RT (right). All the volumes are represented in mean ± standard-error to mean (SEM) (mm^3^). *: p < 0.05; **: p < 0.01; ****: p < 0.0001 (one-way ANOVA). **D** Survival Kaplan–Meier curves were compared between each group using the log-rank test. The sacrifice of mice was determined by the conditions described in the supplementary material and methods. ns: non-significant; **: p < 0.01.

## Data Availability

The datasets supporting the conclusions of this article are included within this article and its additional file. For any further data requests, please contact the corresponding author.

## Abbreviations

AKT: Serine/threonine-protein kinase
ANOVA: Analysis of variance
cDC2: Conventional dendritic cell type 2
CT26: Colon tumor 26 cell line
EGFR: Epithelial growth factor receptor
FoxP3: Forkhead box P3
GDP: Guanosine diphosphate
GTP: Guanosine triphosphate
LL2: Lewis Lung carcinoma cell line
MAPK: Microtubule associated protein kinase
MHC II: Major histocompatibility complex II
NSCLC: Non-small cell lung cancer
ORR: Objective response rate
OS: Overall survival
PD-L1: Programmed cell-death ligand 1
PFA: Paraformaldehyde
PFS: Progression-free survival
PI3K: Phosphatidylinositol 3-kinase
RAS: Rat sarcoma viral oncogene homolog
RT: Radiotherapy
SEM: Standard error to the mean
Tregs: T regulator lymphocytes
WT: Wild-type
