# Supplementary material for: KRASG12C inhibition using MRTX1257: a novel radio-sensitizing partner
Source: J Transl Med. 2023 Oct 31;21:773. doi: 10.1186/s12967-023-04619-0 (PMC10619254; doi:10.1186/s12967-023-04619-0)
Supplement: Supplementary file 1 — Additional file 1: Table S1. List of the antibodies used and their respective dilutions in flow cytometry experiments. Figure S1. (complementary to Fig. 1): MRTX1257 at the concentration of 5 nM or 10 nM for 24 h does not sensitize CT26 KRASG12C+/+ or LL2 NRAS−/− tumor cells to radiation. Clonogenic survival assays were performed in CT26 KRASG12C+/+ or LL2 NRAS−/− tumor cells exposed to various concentrations of MRTX1257 for 24 h. Normalized survival fractions are represented in mean ± standard-error to mean (SEM), with n = 3 to 6 replicates per condition. Survival curves are extrapolations according to the linear quadratic model. Survival curves for A CT26 KRASG12C+/+ cell line and B LL2 NRAS−/− cell line. Figure S2. A single-fraction irradiation of 6 Gy does not increase the efficacy of MRTX1257 in a distant unirradiated tumor. The combination of RT delivered to a single-tumor and oral administration of MRTX1257 was experimented in BALB/c mice bearing bilateral s.c. CT26 KRASG12C+/+ tumors according to the supplementary material and methods. A Schematic view of the experimental setting. A single fraction of 6 Gy was delivered to the right (primary) tumor (primary) whereas the left (secondary) tumor remained unirradiated. B Primary tumor volumes in each condition at the different timepoints (left), and specifically at D6 and D13 after RT (right). All the volumes are represented in mean ± standard-error to mean (SEM) (mm3). *: p < 0.05; **: p < 0.01; ****: p < 0.0001 (one-way ANOVA). C Secondary tumor volumes in each condition at the different timepoints (left), and specifically at D6 and D13 after RT (right). All the volumes are represented in mean ± standard-error to mean (SEM) (mm3). *: p < 0.05; **: p < 0.01; ****: p < 0.0001 (one-way ANOVA). D Survival Kaplan–Meier curves were compared between each group using the log-rank test. The sacrifice of mice was determined by the conditions described in the supplementary material and methods. ns: non-significant; **: p [file 12967_2023_4619_MOESM1_ESM.docx]

**Additional file**

**KRAS^G12C^ inhibition using MRTX1257: a novel radio-sensitizing partner.**

**Pierre-Antoine Laurent^1,2,3*^, Marina Milic^2,3*^, Clément Quevrin^2,3^, Lydia Meziani^2,3^, Winchygn Liu^2,3^, Daphné Morel^4^, Nicolas Signolle^5^, Céline Clémenson^2,3^, Antonin Levy^1,2,3^, Michele Mondini^2,3^, Eric Deutsch^1,2,3^**

***** Equal contributors

Corresponding author: Eric Deutsch, INSERM U1030, 114 rue Edouard Vaillant; 94805 Villejuif, France; eric.deutsch@gustaveroussy.fr

**Supplementary material and methods**

**Dose-response curves on WT cells:**

Two hundred thousand CTT26 WT or LL2 WT cells were seeded in 6-well plates with 2 mL of their respective culture media containing MRTX1257 at various concentrations ranging from 0 nM to 50 µM. At 48 hours of culturing, the medium containing MRTX1257 was changed and washed for a drug-free one. The day after, i.e. at 72h of culturing, cells were harvested separately in each well by using tryspin-EDTA 0.05% (Gicbo) and counted using an automated cell counter Cellometer K2 (Nexcellom Bioscience, MA) after trypan blue staining. The viable cells were thus counted and plotted in dose-response curves.

**Two-tumors experiment**

Immunocompetent female BALB/c mice were subcutaneously inoculated with 1.2 10^6^ CT26 KRAS^G12C+/+^ in their right flank and 8.10^5^ CT26 KRAS^G12C+/+^cells in their left flank, in 50µL of pH 7.2 phosphate-buffer saline (PBS) solution (Gibco).

Once the tumors reached an average of 100-110 mm^3^ for right tumors (primary tumors) and 60-70 mm^3^ for the corresponding left tumors (secondary tumors), estimated using the formula exposed in the methods section, mice were randomized into the different treatment groups.

On the day of randomization (= D-1 before RT), as well as on D1 and D3 after RT, mice received an oral administration of MRTX1257 at the dose of 50 mg/kg reconstituted in 5 ml/kg of Captisol vehicle or the same volume of Captisol vehicle alone, according to the treatment group. At D0, mice randomized into RT only and combination groups received a single fraction of 6 Gy to the primary tumor volume using a Varian Tube NDI 226 (X-ray machine; 200 kV; tube current 15mA; beam filter: 0.2 mmCu, dose-rate 1.15 Gy/minute). Radiation was only delivered to the primary tumor by using a custom shielding and an appropriate device to immobilize the mouse.

Tumor volumes were measured 3 times a week using a digital caliper and the formula described in the methods section. When any of the tumors reached 1200 mm^3^ or when any of critical points including weight loss > 20%, tumor necrosis or suffering appeared, mice were sacrificed.

**Additional file 1: Table: List of the antibodies used and their respective dilutions in flow cytometry experiments.**

**Additional file 1: Figure 1 (complementary to Figure 1): MRTX1257 at the concentration of 5 nM or 10 nM for 24 hours does not sensitize CT26 KRAS^G12C+/+^ or LL2 NRAS^-/-^ tumor cells to radiation.** Clonogenic survival assays were performed in CT26 KRAS^G12C+/+^ or LL2 NRAS^-/-^ tumor cells exposed to various concentrations of MRTX1257 for 24 hours. Normalized survival fractions are represented in mean +/- standard-error to mean (SEM), with n=3 to 6 replicates per condition. Survival curves are extrapolations according to the linear quadratic model. Survival curves for (**A**) CT26 KRAS^G12C+/+^ cell line and (**B**) LL2 NRAS^-/-^ cell line.

**Additional file 1: Figure 2: A single-fraction irradiation of 6 Gy does not increase the efficacy of MRTX1257 in a distant unirradiated tumor.** The combination of RT delivered to a single-tumor and oral administration of MRTX1257 was experimented in BALB/c mice bearing bilateral s.c. CT26 KRAS^G12C+/+^ tumors according to the supplementary material and methods. (**A**) Schematic view of the experimental setting. A single fraction of 6 Gy was delivered to the right (primary) tumor (primary) whereas the left (secondary) tumor remained unirradiated. (**B**) Primary tumor volumes in each condition at the different timepoints (left), and specifically at D6 and D13 after RT (right). All the volumes are represented in mean +/- standard-error to mean (SEM) (mm^3^). *: p<0.05; **: p<0.01; ****: p<0.0001 (one-way ANOVA). (**C**) Secondary tumor volumes in each condition at the different timepoints (left), and specifically at D6 and D13 after RT (right). All the volumes are represented in mean +/- standard-error to mean (SEM) (mm^3^). *: p<0.05; **: p<0.01; ****: p<0.0001 (one-way ANOVA). (**D**) Survival Kaplan-Meier curves were compared between each group using the log-rank test. The sacrifice of mice was determined by the conditions described in the supplementary material and methods. ns: non-significant; **: p<0.01.

Additional file 1: TABLE

| Antibody (fluorochrome) | Clone | Manufacturer | Dilution |
| --- | --- | --- | --- |
| CD 16/32 Fc Block | 93 | BioLegend | 1:50 |
| CD45 (APC Vio770) | REA 737 | Miltenyi Biotec | 1:50 |
| CD11b (BUV395) | M1/70 | BD Biosciences | 1:100 |
| CD11b (BV605) | M1/70 | BioLegend | 1:100 |
| PD1 (PerCp Vio700) | REA 802 | Miltenyi Biotec | 1:50 |
| PD-L1 (BV650) | MIH5 | BD Biosciences | 1:100 |
| CD4 (BV510) | RM 4-5 | BioLegend | 1:400 |
| CD8 (BUV737) | 53-6.7 | BD Horizon | 1:400 |
| NKp46 (BV421) | 29A 1.4 | BioLegend | 1:100 |
| CD64 (BV605) | X 54-5/7.1 | BioLegend | 1:50 |
| Ly6G (PerCp Vio700) | REA 526 | Miltenyi Biotec | 1:50 |
| CD11c (PE Vio770) | REA 754 | Miltenyi Biotec | 1:50 |
| H-2Ld/H-2Db (BV650) | 28-14-8 | BD Optibuild | 1:100 |
| Ly6c (AlexaFluor700) | HK1.4 | BioLegend | 1:50 |
| MHC II (VioGreen) | REA 813 | Miltenyi Biotec | 1:50 |
| FoxP3 (PE) | REA 788 | Miltenyi Biotec | 1:50 |
| CD80 (PE Vio615) | REA 983 | Miltenyi | 1:50 |

**
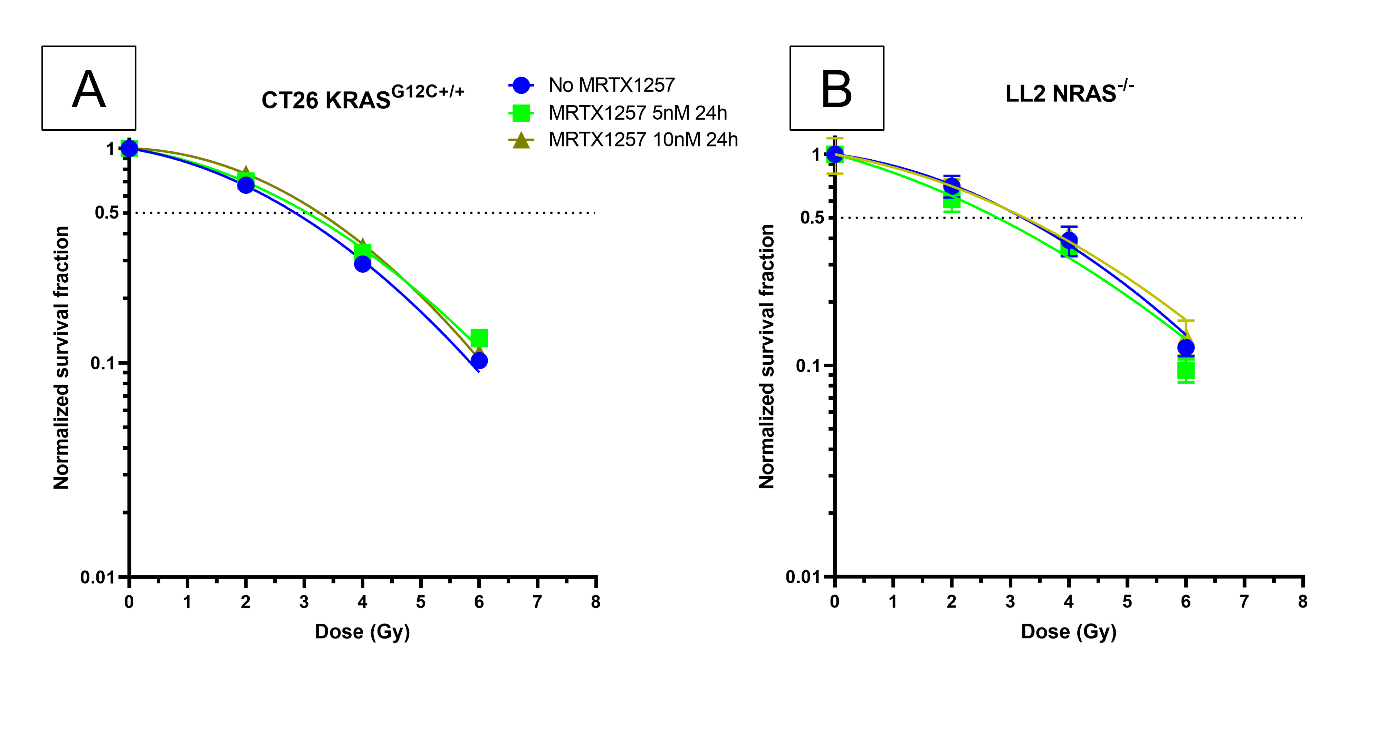
**

Additional file 1: FIGURE 1


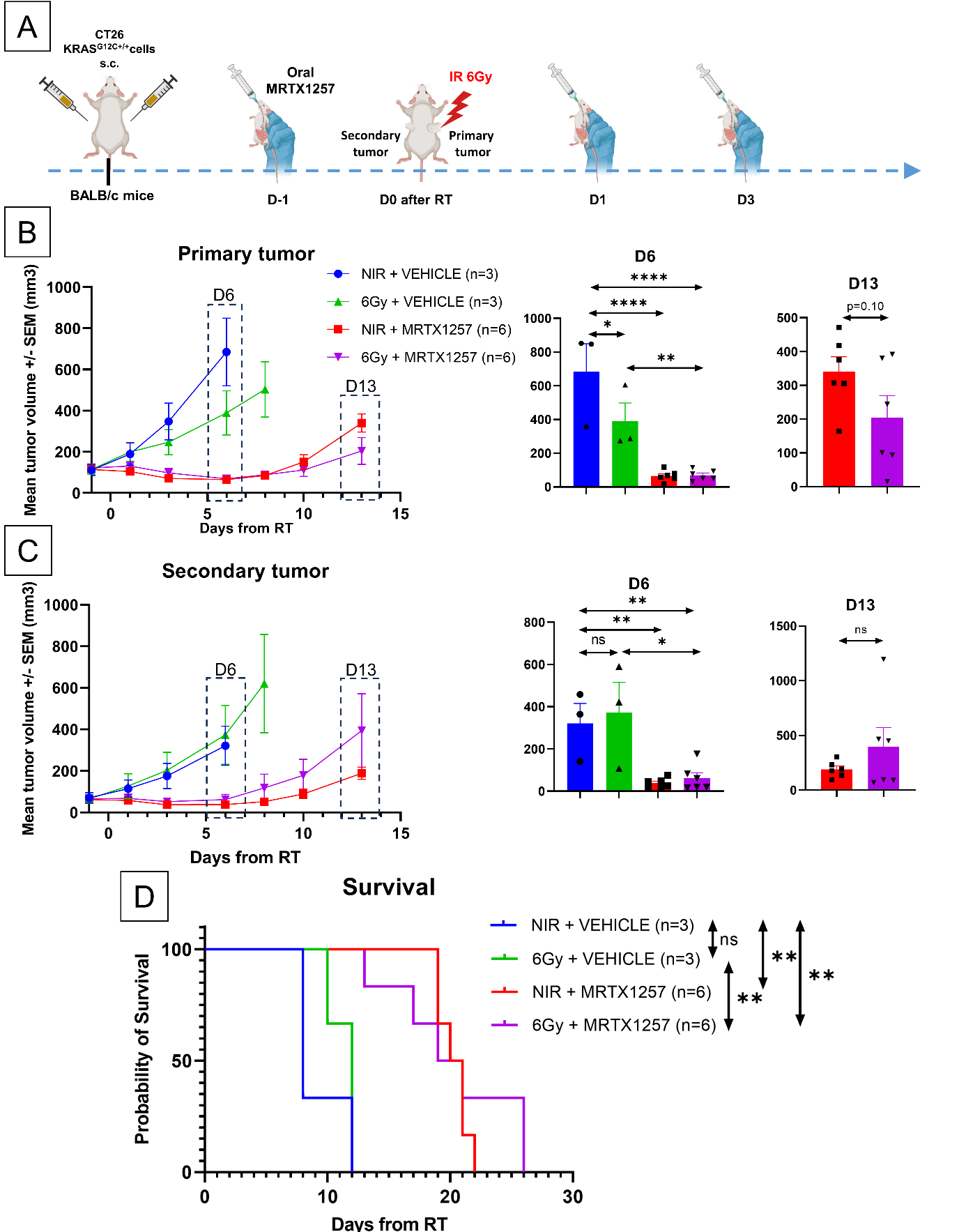


Additional file 1: FIGURE 2

s
